# Chronic Astrocytic TNFα Production in the Preoptic-Basal Forebrain Causes Aging-like Sleep–Wake Disturbances in Young Mice

**DOI:** 10.3390/cells13110894

**Published:** 2024-05-22

**Authors:** Andrey Kostin, Md. Aftab Alam, Anton Saevskiy, Md. Noor Alam

**Affiliations:** 1 Research Service (151A3), Veterans Affairs Greater Los Angeles Healthcare System, Sepulveda, CA 91343, USA; andrey.kostin@usa.com (A.K.); aftabalam@ucla.edu (M.A.A.); 2 Department of Psychiatry, University of California, Los Angeles, CA 90025, USA; 3 Scientific Research and Technology Center for Neurotechnology, Southern Federal University, 344006 Rostov-on-Don, Russia; saevskiy@sfedu.ru; 4 Department of Medicine, University of California, Los Angeles, CA 90025, USA

**Keywords:** preoptic area, basal forebrain, inflammation, sleep–wake disturbances, TNFα, astrocytes

## Abstract

Sleep disruption is a frequent problem of advancing age, often accompanied by low-grade chronic central and peripheral inflammation. We examined whether chronic neuroinflammation in the preoptic and basal forebrain area (POA-BF), a critical sleep–wake regulatory structure, contributes to this disruption. We developed a targeted viral vector designed to overexpress tumor necrosis factor-alpha (TNFα), specifically in astrocytes (AAV5-GFAP-TNFα-mCherry), and injected it into the POA of young mice to induce heightened neuroinflammation within the POA-BF. Compared to the control (treated with AAV5-GFAP-mCherry), mice with astrocytic TNFα overproduction within the POA-BF exhibited signs of increased microglia activation, indicating a heightened local inflammatory milieu. These mice also exhibited aging-like changes in sleep–wake organization and physical performance, including (a) impaired sleep–wake functions characterized by disruptions in sleep and waking during light and dark phases, respectively, and a reduced ability to compensate for sleep loss; (b) dysfunctional VLPO sleep-active neurons, indicated by fewer neurons expressing c-fos after suvorexant-induced sleep; and (c) compromised physical performance as demonstrated by a decline in grip strength. These findings suggest that inflammation-induced dysfunction of sleep- and wake-regulatory mechanisms within the POA-BF may be a critical component of sleep–wake disturbances in aging.

## 1. Introduction

Sleep disruption is one of the most frequent and challenging problems of advancing age. The hallmarks of human sleep in aging include difficulty falling asleep, sleep disruption by frequent awakenings, decreased non-rapid eye movement (nonREM) sleep, and its slow wave activity (SWA) [1,2,3]. Other features include a decline in REM sleep, a dampening of the sleep/wake circadian rhythm amplitudes, and a decline in homeostatic response to sleep loss [4,5]. Aging is also associated with daytime sleepiness and shortening of sustained waking bouts [2,3]. In rodents, aging is associated with similar changes in sleep–wake (S–W) organization [6,7,8,9]. However, the degree of sleep disruption in aging shows large inter-individual variability, indicating that other processes or factors that interact with aging potentially contribute to the severity of aging-associated decline in sleep quantity and quality [3].

Another hallmark feature of aging is a low-grade chronic central and peripheral inflammation. Both animal and human studies indicate that the brains of older subjects are in a native heightened inflammatory state, even in the absence of overt disease [10,11]. Aging is also characterized by systemic chronic inflammation [12]. Much evidence suggests that chronic inflammation plays a central role in cellular aging, including neuronal aging, and may be detrimental to various CNS functions [11,12,13]. For example, chronic systemic inflammation induces telomere dysfunction, a decline in neurogenesis, and accelerates cell senescence in the absence of any apparent genetic or environmental factors [8,12,14,15,16,17]. Chronic neuroinflammation has also been implicated in the development and progression of neurodegenerative diseases [12,18]. And people with neurodegenerative diseases, e.g., Alzheimer’s disease, and Parkinson’s disease, often experience sleep–wake disturbances, including features of sleep–wake disruption experienced by the aging population [19,20,21].

Multiple lines of studies support that the preoptic area (POA), especially its ventrolateral preoptic region (VLPO), is a critical sleep-regulatory structure [22,23,24]. VLPO lesions cause sleep fragmentation and insomnia [25], whereas selective activation of VLPO neurons projecting to the hypocretin field promotes sleep [26]. VLPO neurons exhibit sleep-associated c-fos expression (Fos-IR) and increased Ca^2+^ activity, markers of neuronal activation during nonREM and or REM sleep [27,28,29]. The VLPO sleep-active neurons typically exhibit increasing discharge from waking → nonREM sleep transition → stable nonREM/REM sleep [30,31]. Their activity profiles further suggest that VLPO sleep-active neurons are involved in responding to homeostatic sleep need [29,31]. While VLPO contains a high concentration of sleep-active neurons, these neurons are diffusely distributed in the VLPO core and its adjoining POA areas and are mixed with wake-active neurons [32,33,34]. The basal forebrain (BF) adjacent to the POA has been implicated in the regulation of cortical activation, arousal, and attention [35,36,37,38]. The BF consists of three major neurochemically distinct populations of cortically projecting and wake-promoting neurons containing acetylcholine, GABA, especially those containing the calcium-binding protein parvalbumin, and glutamate, although a relatively smaller population of sleep-active neurons have also been reported [36,39,40]. The features of sleep–wake disturbances in aging suggest a weakening or dysfunction of both sleep- and wake-regulatory systems. However, what contributes to the dysfunction of sleep- and wake-regulatory systems in aging remains poorly understood.

We hypothesize that chronic inflammation in critical sleep- and wake-regulatory systems contributes to their dysfunction, leading to sleep–wake disturbances in aging. In this study, to evaluate this hypothesis, we induced chronic neuroinflammation in the POA-BF of young mice by selectively triggering tumor necrosis factor-alpha (TNFα) production in astrocytes and assessed its impact on (a) 24 h spontaneous sleep–wake organization, especially on features of sleep–wake disturbances; (b) homeostatic responses to acute and short-term sleep loss; and (c) the responsiveness of VLPO neurons to pharmacologically induced sleep. Additionally, we examined whether sleep–wake disruption affected overall frailty by evaluating motor and physical performance. To subject the sleep- and wake-regulatory neuronal groups in the POA-BF to chronic neuroinflammation, we designed a viral vector to express TNFα exclusively in the astrocytes within the POA-BF, thus creating an extracellular inflammatory environment for local neurons through continuous TNFα production. TNFα is recognized for its pleiotropic role in immune responses and inflammatory reactions [41,42,43,44], and astrocytes are one of the most abundant cell types in the CNS that produce TNFα under both physiological and pathological conditions [45,46,47].

## 2. Materials and Methods

TNFα is a cytokine and a central regulator of inflammatory responses. We determined the effects of chronic viral vector-mediated TNFα production in astrocytes localized in the POA-BF on sleep–wake parameters, sleep-regulatory challenges, sleep-associated Fos-IR in VLPO neurons, and locomotor and gross motor functions in young mice.

### 2.1. Experimental Subjects

Experimental subjects were 17 male and 17 female C57BL6 wild-type mice that were 3–4 months old and weighed 24–28 g at the time of surgery. Of these, 3 mice could not be used due to mechanical failures. These mice were maintained on a 12 h:12 h light/dark cycle (lights on at 8:00 h), an ambient temperature of 24 ± 2 °C, and with food and water available ad libitum. All experiments were conducted in accordance with the National Research Council’s “Guide for the Care and Use of Laboratory Animals” and were approved by the Institutional Animal Research Committee of the Veterans Affairs Greater Los Angeles Healthcare System.

### 2.2. Viral Vector

Our objective was to expose sleep- and wake-active neuronal groups in the POA-BF to chronic neuroinflammation via transgenic overproduction of TNFα within astrocytes and into the extracellular environment. To accomplish this, we developed an AAV-based viral vector incorporating the gene of interest into the AAV5 serotype envelope, known for its heightened affinity for transfecting astrocytes along with the GFAP promoter to drive *TNFα* gene expression in astrocytes [48,49]. The resulting construct, AAV5 carrying the *TNFα* gene under the control of the GFAP promoter, was further supplemented with an additional gene for bicistronic expression of mCherry protein, serving as a marker for TNFα expression. We expected that by employing this custom-designed AAV5-GFAP-TNFα-mCherry construct, we could selectively transfect local astrocytes with *TNFα-mCherry* genes within the POA-BF, resulting in sustained TNFα protein expression and subsequent chronic inflammation around the vector injection site.

### 2.3. Surgical Procedures

The details of the general surgical procedures have been described previously [6,50]. Briefly,

(i) Viral vector injection: Under surgical anesthesia (100 mg/Kg ketamine + 15 mg/Kg xylazine; maintenance with isoflurane) and aseptic conditions, mice were placed in a stereotaxic frame, the skull was exposed and cleaned, and a hole (~1 mm in diameter) was drilled unilaterally in the skull just above the VLPO. Then, the needle (30 G) of a micro-syringe (Hamilton Co., Reno, NV, USA) loaded with viral vector and attached to a motor-driven micromanipulator was inserted through the hole into the VLPO (AP: 0.0–0.1 mm, ML: 0.5 mm from bregma, and DV: 5.5–5.6 mm from the scalp) [26,51] for delivering viral vectors. Mice were injected with 0.1, 0.2, and 0.4 µL (titer ≥ 2.3 × 10^13^ vector genomes per mL) of AAV5-GFAP-TNFα: IRES: mCherry:WPRE (customized, VectorBuilder Inc., Chicago, IL, USA) or comparable volume of its EGFP control viruses AAV5-GFAP:IRES:mCherry:WPRE (Addgene, Watertown, MA, USA) into the VLPO. For control, we used only 0.2 and 0.4 µL of the control vector. The viral vectors were injected slowly over a 10–25 min period (0.1 µL/5 min). After injection, the needle was left in place for another 20 min and then slowly withdrawn to avoid any backflow.

(ii) Implantation of EEG and EMG electrodes: Mice were implanted with electroencephalogram (EEG) and electromyogram (EMG) electrodes for polygraphic monitoring of sleep–waking states. For unilateral bipolar EEG recording, two stainless steel screw electrodes were implanted 1 mm rostral and 1 mm lateral to bregma, and 4 mm posterior and 2 mm lateral to bregma. Two flexible silver stainless steel EMG wire electrodes were implanted into the dorsal cervical musculature. The ends of the EEG and EMG electrodes were then soldered with a small and lightweight connector, which was fixed to the skull with dental cement. The open wounds were sutured, and the mice were returned to their home cages.

### 2.4. Recovery and Adaptation

Mice were allowed to recover from the surgical procedure for 8–10 days in Plexiglas recording cages placed in a sound-attenuated, temperature-controlled recording chamber. The mice were then connected to the recording cable, which allowed an additional 2–3 days for acclimatization to the recording setup, recording cables, and experimental handling. In the recording chamber, the mice were maintained at the same 12 h:12 h light: dark cycle, an ambient temperature of 24 ± 2 °C, and with ad libitum access to food and water.

### 2.5. Data Acquisition

We conducted the following two sets of studies on the same cohort of animals:

A. Sleep–wake recording: After postsurgical recovery and adaptation to the recording setup, sleep–wake profiles of mice were recorded continuously for four weeks, starting week 2, after vector injection. This allowed us to record sleep–wake profiles of animals with progressively increasing expression of TNFα in the astrocytes and consequent local inflammatory milieu around the neuronal population in the vector’s diffusion field within the POA-BF.

At the end of 4 weeks of recording, the effects of homeostatic response to 3 h of sleep deprivation (SD) were assessed. Mice were sleep-deprived using a gentle handling and environmental enriching procedure, which included the introduction of new objects, e.g., small balls or plastic bones, or new nesting material, e.g., cotton or paper shreds into the cage in order to keep the animals occupied and replacing them with new ones when animals appeared becoming drowsy, or tapping on the cage or gently blowing with a small dropper-like blower near them [50]. SD started right after light onset. Following SD, mice were left undisturbed, and their sleep–wake profile was recorded for another 9 h. Since homeostatic recovery is robust during the first 2–3 h even after 6 h of SD, we divided 9 h of the post-deprivation period into 2 blocks of 3 h and 6 h for analysis.

The amplified and filtered EEG and EMG signals were continuously digitized and stored on the computer’s hard disk using an integrated computer interface device (Cambridge Electronic Design 1401, Cambridge, UK; supporting software, Spike 2, Version 6) for subsequent sleep–wake scoring and analyses.

B. Behavioral testing: After recording sleep–wake parameters, control, and TNFα mice were disconnected from the recording setup and tested for general locomotor activity and motor functions. The following tests were conducted using standard protocols.

(i) The Open-Field test: for determining the general activity levels, gross locomotor activity, and exploration habits [52,53,54]. Briefly, animals were left in an open-field arena (40 × 40 × 20 inches) for 10 min, and their activity was video-recorded and analyzed using an automated tracking system ANY-maze for various parameters, including maximum distance traveled, velocity, movement duration, and time spent in pre-defined zones.

(ii) Rotarod test: for assessing sensorimotor coordination and motor learning [55,56]. The mice were placed on a rotating rod and underwent 3 initial training sessions of 60 s each (1 at 0 rpm and 2 at 4 rpm). After training, mice were placed on the rotating rod set to accelerate from 0 to 40 rpm in 300 s, and the ability of mice to remain on the rod or the latency of fall from the rod, and whether animals clung to the rod and completed full passive rotation, were recorded.

(iii) Wire hang test: for evaluating deficits in motor functioning, particularly muscle strength, endurance, and grip strength [57,58]. The mice were placed on a wire grid, which was then turned upside down, and the latency to fall or the ability to maintain the grip was recorded. One session consisting of a set of three trials was conducted on each animal.

C. Effects of suvorexant: After the behavioral assessment, by the end of the 6th week after vector injection, both TNFα and control mice were reconnected and, after one day of acclimatization, were injected with suvorexant (30 mg/kg, IP) at the end of the light period. Suvorexant is a selective dual orexin receptor antagonist known for its potent sleep-inducing effects in both humans and animals [59,60]. While 10–100 mg/kg of suvorexant has been used in sleep studies in mice, we used 30 mg/kg because this dose significantly reduces waking and increases nonREM and REM sleep during the first 2 h after administration [61], a timeline which was used to detect sleep-associated fos expression in this study. 5 mg of suvorexant (provided by NIDA Drug Supply Program) was dissolved in 1 mL of a 30% solution of cyclodextrin and administered just prior to dark onset when sleep pressure is minimal in both TNFα and control mice. We used suvorexant, known to inhibit wake-promoting neurons, at the dark onset to evoke sleep-associated c-fos expression in sleep-active neurons and use it to quantify dysfunctional sleep-active neurons in the VLPO in TNFα vs. control mice. Suvorexant-treated mice were sacrificed after 2 h of pharmacologically enhanced sleep opportunity. Typically, 90–120 min of recovery sleep after SD has been used to identify neurons exhibiting sleep-associated Fos-IR or sleep-active neurons in VLPO, which may also include wake-active neurons with residual Fos-IR [27,28].

### 2.6. Perfusion and Histological Processing

Mice were deeply anesthetized (100 mg/kg, IP, pentobarbital) and perfused transcardially with phosphate-buffered saline (PBS, 0.1 M, pH 7.2), followed by 4% paraformaldehyde in PBS [6,26]. Brains were extracted, placed into 30% sucrose solution until they sank, and cut coronally at 30 µm using a freezing microtome. Coronal sections through the VLPO were immuno-stained for visualization of ionized calcium-binding adapter molecule 1 (Iba1; ab107159, 1:800; Abcam, Waltham, MA, USA), neuronal nuclear antigen (NeuN; ab104225, 1:1000; Abcam), and suvorexant-induced Fos-IR (AB-5, 1:15,000; Oncogene Science, Uniondale, NY, USA) in VLPO neurons using standard protocols [26,27]. However, coronal sections spanning from the basal forebrain through the posterior hypothalamic area were used for visualization of injection sites and the spread of mCherry. Sections were mounted and cover-slipped using a fluorescence mounting medium (ProLong™ Gold Antifade Mountant, P36931, Thermo Fisher Scientific, West Hills, CA, USA). The fluorescent images were acquired using an LSM 900 Zeiss confocal microscope (Zeiss, Maple Grove, MN, USA).

### 2.7. Data Analyses

A. Sleep–wake analyses: The EEG and EMG data files were numbered so that data could be processed and analyzed in a blind manner. Sleep–wake profiles of animals were scored in 10 s epochs in terms of waking, nonREM sleep, and REM sleep using our in-house Python-based (Version 3.12) software based on EEG spectrum and further verified randomly against manual analysis using the standard criteria [50]. The continuity and stability of sleep–wake states in control and TNFα groups was further determined by classifying the episode duration of each of the 3 sleep–waking stages into 4 subcategories: episodes with a duration of 0–120 s (short), >2 min to 5 min (medium), >5 to 10 min (long), and >10 min (extended). Each week of analysis included average data of three days of sleep–wake recording (3 × 24 h/mouse).

EEG spectral analysis was performed using Fast Fourier Transform for nonREM sleep delta (FFT; 1.0–4.0 Hz, further sub-classified into low-delta, 1–2 Hz, and high-delta, 3–4 Hz) and waking theta (5–9 Hz). Delta power in nonREM sleep during the recovery period was averaged for the first 3 h followed by the next 6 h and normalized against average delta power in baseline nonREM sleep during the same time points in TNFα and control mice. The power of raw theta was evaluated in all artifact-free epochs of waking during SD in TNFα and control mice.

The open-field data were analyzed using the ANY-maze video tracking software (Version 7.4; Stoelting Co. Wood Dale, IL, USA). The rotarod and hanging data were plotted manually.

B. Confocal imaging and cell counting: The number of c-fos+ cells, NeuN+ cells, Iba1+ cells, and mCherry+ cells were visualized and analyzed on ipsilateral and contralateral sides in TNFα and control groups using an LSM 900 Zeiss confocal microscope and ImageJ software (https://imagej.net/software/fiji, version 1.53p). Because of the morphological complexity of activated Iba1 cell forms, we assessed exclusively de-ramified cells, i.e., cells devoid of branches and small amoeboid shapes, which serve as markers of strong inflammation [62,63,64].

C. Statistical analyses: The SigmaPlot (version 12; Systat Software, San Jose, CA, USA) software package was used for statistical analyses of the data. The data are presented as mean ± standard error of the mean (SEM). One-way repeated-measures analysis of variance (ANOVA) for within-group analysis, followed by multiple pairwise comparisons using the Student–Newman–Keuls method, was used for comparing sleep–wake changes at different time points in various TNFα or control groups. In a few cases, the data failed normality tests. Those data points were analyzed using equivalent non-parametric Friedman RM ANOVA on ranks. The sleep–wake changes or changes in various immunohistochemical parameters between control and TNFα groups were compared using one-way ANOVA for more than two groups and the Student’s *t*-test or Mann–Whitney rank sum test in the case where the normality test failed for two groups.

## 3. Results

### 3.1. TNF-α Expression in the POA-BF

**Figure 1:**
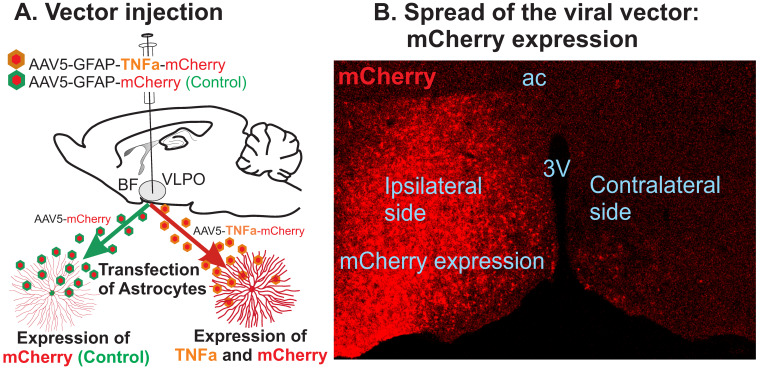
Schematic of the procedure used for inducing TNFα expression/production in the astrocytes within the VLPO-BF. (**A**) Schematic representation of the injections of viral vector AAV5-GFAP-TNFα-mCherry or its control virus, AAV5-GFAP-mCherry, in the VLPO and potential outcomes of this approach. (**B**) Photomicrograph of a representative histological section showing Cre-dependent expression of mCherry and its spread in the POA-BF region. The mCherry expression, which was used to identify the injection and spread of the viral vector, was mostly limited to the ipsilateral side; still, in a few cases, it spread to the contralateral side on the rostral end without much of the ventricular barrier. 3V = third ventricle; AC = anterior commissure.

The injection sites and spread of the viral vector were verified by the expression of mCherry under the control of the GFAP promoter. Figure 1 shows the injection schematic and a representative spread of mCherry expression six weeks post-vector injection. In both the control and TNFα groups, the injected vector diffused across a broader area (approximately 1–2 mm, depending on volume) and transfected the VLPO, medial POA, dorsolateral POA, and parts of the BF, including the horizontal limb of the diagonal band, septum, and cholinergic magnocellular areas. TNFα mice, compared to control, demonstrated significant changes in their sleep/wake profile. Thus, it is likely that the observed changes in sleep–wake patterns in this study were caused by elevated TNFα production, primarily within the POA and the BF. Also, the spread of the viral vector in the control and TNFα groups were broadly comparable. Thus, it is likely that comparable populations of POA-BF astrocytes and neurons were affected in TNFα and control groups. Importantly, animals were euthanized 1–2 weeks after the presented sleep–wake data. Therefore, the possibility that the observed levels of microglia activation and resulting neuroinflammatory environment within the POA-BF might have contributed to even more pronounced changes in sleep–wake dynamics cannot be ruled out.

**Figure 2:**
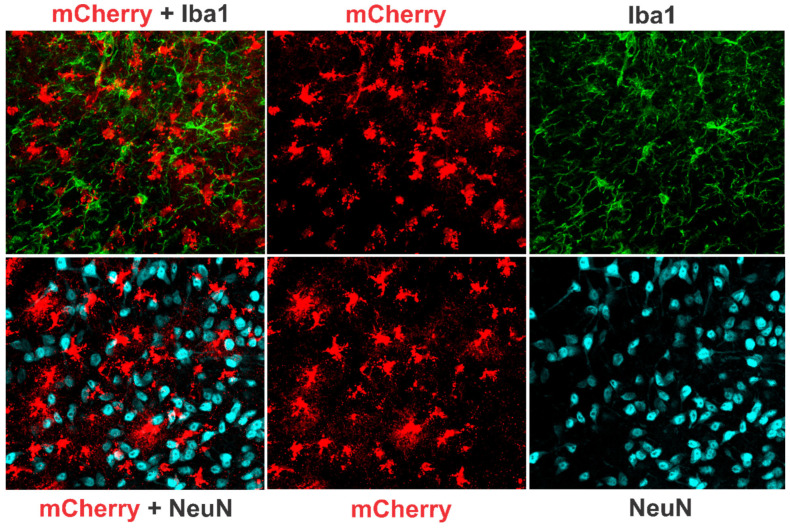
Photomicrographs of representative histological sections showing mCherry expression among NeuN+ and Iba1+ cells after AAV5-mCherry injection. An exclusive expression of mCherry can be seen in astrocyte-like cells. While mCherry expression was extensive among NeuN+ or Iba1+ cells, no colocalization of mCherry with either NeuN+ or Iba1+ cells was observed. mCherry expression, however, was predominantly colocalized with GFAP+ cells (see Figure S1).

Furthermore, immunohistochemical analysis revealed no colocalization of mCherry with NeuN+ or Iba1+ cells (Figure 2), but predominantly with GFAP+ cells (Supplementary Figure S1), indicating that vector-mediated TNFα expression/production was specifically confined to astrocytes localized in the vector’s diffusion field.

### 3.2. Effects of Chronic TNFα Expression/Production in the POA-BF on Sleep–Wake Architecture

The sleep–wake profiles of TNFα and control mice were compared at the end of 2, 3, 4, and 5 weeks after viral vector injection.

#### 3.2.1. Effects of AAV5-GFAP-mCherry Injections (Control Group) in the POA-BF on Sleep–Waking

**Figure 3:**
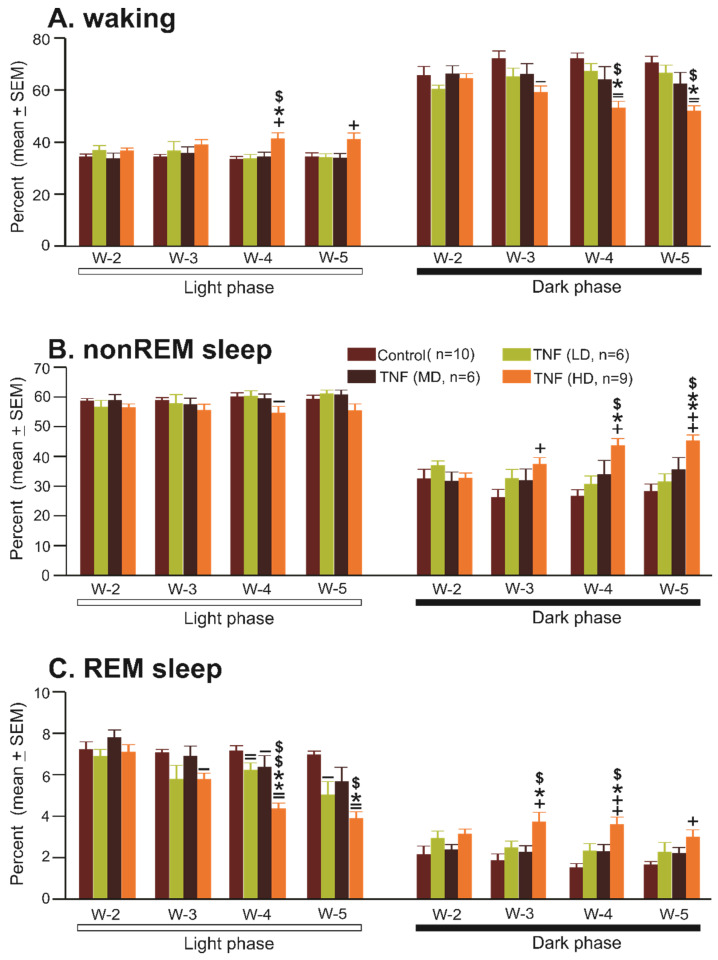
Chronic TNFα production in the POA-BF causes sleep–wake disturbances. Percent time spent (mean ± SEM) by control and TNFα groups of mice in waking, nonREM sleep, and REM sleep at the end of post-injection week 2 (W-2), week 3 (W-3), week 4 (W-4), and week 5 (W-5) during the light and dark period. The TNFα caused a significant increase in waking and a decrease in sleep during the light phase and opposite effects, i.e., decreased waking and increased sleep during the dark phase, indicating that TNFα production affected both sleep- and wake-promoting systems. The effect was largely post-treatment time and dose dependent. +, −, increase and, decrease compared to control group, *, compared to low dose (LD), $, compared to medium dose (MD). +, −, *, $, <0.05, ++, =, **, $$, *p* < 0.01 level of significance (one-way ANOVA followed by Student–Newman–Keuls method for multiple comparison).

The sleep–wake profiles of mice at 2, 3, 4, and 5 weeks after AAV5-GFAP-mCherry injection (*n* = 10) are shown in Table 1, Figure 3. The amounts of waking, nonREM sleep, and REM sleep in the control group at 2 weeks, 3 weeks, 4 weeks, and 5 weeks after the control virus injection were comparable during both light and dark phases. These mice spent a significant portion of the light phase in sleep and were predominantly awake during the dark phase. Thus, mice with AAV5 injection or mCherry expression in the astrocytes in the POA-BF seem to exhibit sleep–wake organization or circadian expression similar to those exhibited by typical untreated mice of this age group, which did not change with the dose/volume of the vector used or the progression of vector expression with time.

#### 3.2.2. Effects of AAV5-GFAP-TNFα-mCherry Injections (TNFα Group) in the POA-BF on Sleep–Waking

Table 1 and Figure 3 show the effects of various doses/volumes of AAV5-GFAP-TNFα-mCherry injections on the sleep–wake profiles of mice at the end of 2, 3, 4, and 5 weeks after treatments. Mice in TNFα groups exhibited a time-after-treatment and dose-dependent effect on the amounts of waking, nonREM sleep, and REM sleep both during the light and dark phases.

As a group, mice treated with low and medium doses of TNFα vector (TNF-V) exhibited largely comparable amounts of waking, nonREM sleep, and REM sleep after 2, 3, 4, and 5 weeks, except for minor changes in REM sleep during the light phase. However, mice treated with the higher dose of TNF-V exhibited significant changes in sleep–waking. The onset of effects started after week (W) 3, and changes became more apparent in W4 and W5.

1. Sleep–wake amounts

In general, mice treated with the higher dose of TNF-V exhibited opposite changes in sleep–wake architecture during the light and dark phases (Figure 3).

During the light phase, compared to the control group, mice treated with the higher dose of TNF-V exhibited significant increases in waking during W4 and W5. On the contrary, REM sleep exhibited the strongest percent decline, which started during W3 and worsened during W4 and W5 (Table 1, Figure 3). NonREM sleep exhibited a general decline, which was significant only during W4.

During the dark phase, mice treated with the higher dose of TNF-V exhibited a significant decline in waking compared to control and those treated with smaller and medium doses of TNF-V, especially during W4 and W5. On the contrary, both nonREM sleep and REM sleep exhibited a general and, to some extent, progressive increase, starting W3 and continuing through W5 (Table 1, Figure 3).

2. Sleep–wake stability

**Figure 4:**
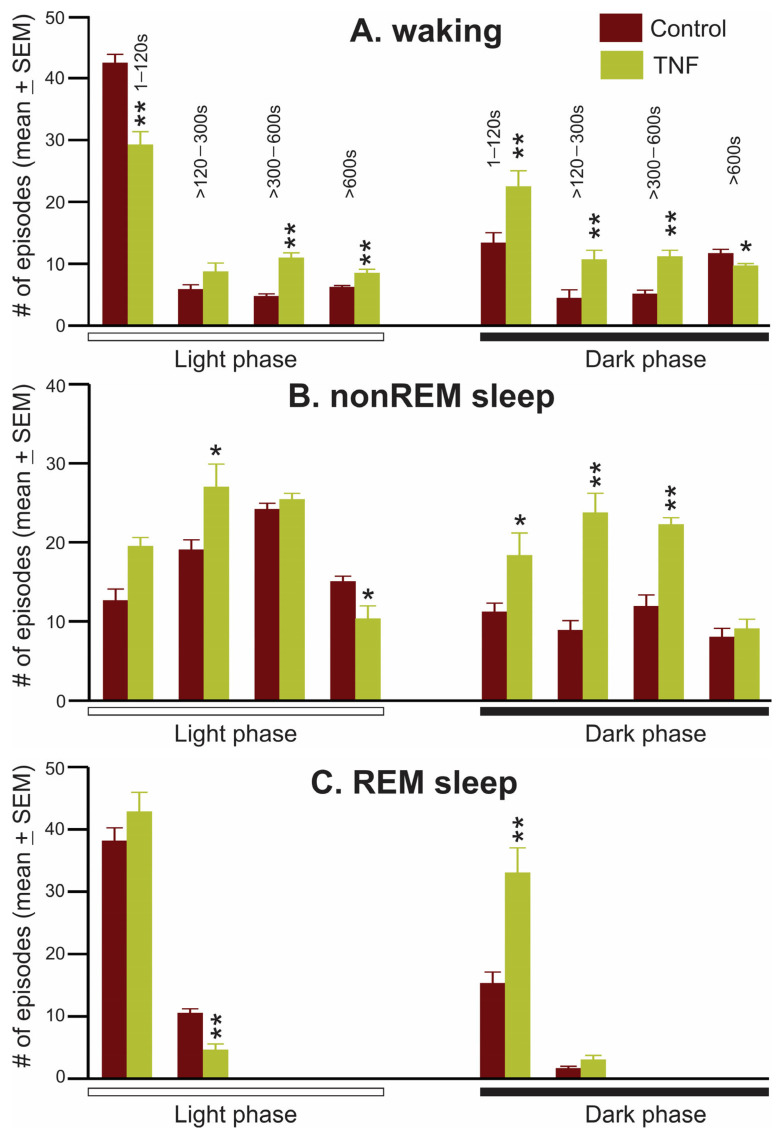
Chronic TNFα production in the VLPO-BF causes sleep–waking instability. Number (#, mean ± SEM) of episodes of waking, nonREM sleep, and REM sleep of various durations exhibited by control and TNFα mice 5 weeks post-treatment. Compared to the control, TNFα mice exhibited more fragmented waking during the dark phase and fragmental nonREM and REM sleep during the light phase. During the light phase, TNFα mice showed a significant increase in the long and extended bouts of waking and a decrease in the number of extended bouts of nonREM and medium bouts of REM sleep. On the contrary, during the dark phase, these mice exhibited a significant increase in the small–medium and a decline in longer bouts of waking, and significant increases in the number of small–large episodes of nonREM and REM sleep. * *p* < 0.05; ** *p* < 0.01; independent *t*-test or Mann–Whitney rank sum test, in case normality test failed.

Figure 4 shows a comparison of the frequencies of waking, nonREM sleep, and REM sleep episodes of different durations encountered in control and TNFα mice. As changes in sleep–wake amounts were most pronounced five weeks post-TNF-V treatment, we analyzed waking, nonREM sleep, and REM sleep episodes on W5 sleep–wake data from control and TNFα mice to determine if TNFα production induced sleep–wake instability.

During the light phase, compared to control, TNFα mice exhibited significantly higher sleep instability as marked by: (a) higher numbers of short (NS, *p* = 0.056) to medium episodes of nonREM sleep and a decrease in extended episodes of nonREM sleep; (b) significantly fewer episodes of REM sleep of medium duration (120–300 s) and increased REM episodes of shorter duration; and (c) more stable waking, as marked by an increased number of long and extended episodes and decrease in the number of shorter episodes of waking (Figure 4).

Conversely, during the dark phase, compared to control, TNFα mice exhibited significantly higher waking instability as marked by: (a) an increase in the frequency of short to medium episodes and a decrease in extended waking episodes; (b) increased sleep intrusion as marked by a significant increase in the occurrence of shorter episodes of nonREM sleep; (c) more stable nonREM sleep as marked by an increased number of medium and longer episodes of nonREM sleep; and (d) an increased number of REM sleep episodes of 0–120 s (Figure 4).

3. Homeostatic sleep response

**Figure 5:**
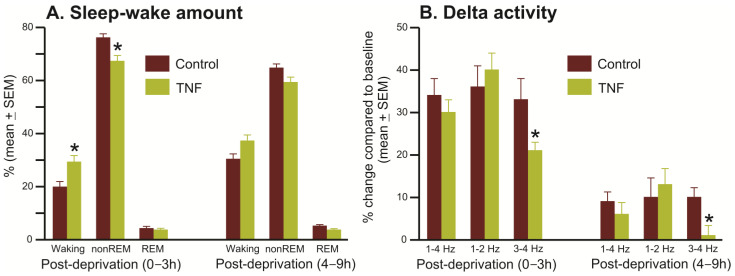
TNFα mice exhibit a poor homeostatic response to sleep deprivation. (**A**) Time spent (%, mean ± SEM) in waking, nonREM sleep, and REM sleep during the 9 h (divided into first 3 and last 6 h) recording after 3 h of SD in control (*n* = 6) and TNFα (*n* = 9) mice. The TNFα mice spent significantly more time in waking and less time in nonREM sleep during the first 3 h of the recovery period. (**B**) % change compared to the baseline in delta activity during nonREM recovery sleep post-SD. TNFα mice exhibited significantly lower levels of high-delta activity in nonREM sleep during the recovery period. * *p* < 0.05; independent t-test or Mann–Whitney rank sum test, in case normality test failed.

**Figure 6:**
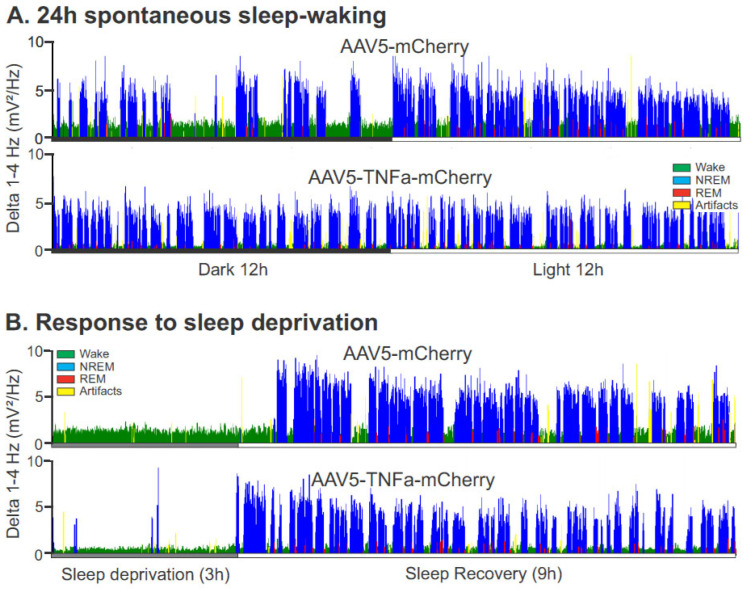
TNFα mice exhibit lower delta power during spontaneous sleep and recovery sleep after SD. (**A**) Continuous recording showing EEG spectral power in the delta range (1–4 Hz) across 24 h of spontaneous sleep–wake recording in individual control and TNFα mice. In TNFα mice, a fragmented waking state with increased sleep intrusions during the dark phase can be observed. Additionally, highly fragmented nonREM sleep with lower delta power during the light phase is also evident. (**B**) TNFα mice also exhibited lower sleep consolidation and delta power during recovery sleep after 3 h of SD.

We compared sleep–wake architecture during 0–3 h and 4–9 h of the recovery period after 3 h of SD in control (*n* = 6) and TNFα (*n* = 9) mice to determine if TNF-V-treated animals exhibited any deficit in homeostatic sleep response. The sleep–wake architecture during the recovery period after 3 h of sleep deprivation in control and TNFα mice is shown in Figure 5 and Figure 6.

During 0–3 h of the recovery period, compared to control, TNFα mice exhibited significantly reduced nonREM sleep rebound and increased levels of arousal (Figure 5A and Figure 6B). During the 4–9 h of the recovery period, while waking, nonREM, and REM sleep amounts were comparable in both groups, TNFα mice exhibited relatively higher levels of waking (37 vs. 30%, t = 2.07, *p* = 0.058) and decreased nonREM sleep (59 vs. 65%, t = 1.90; *p* = 0.079). The amount of REM sleep in the recovery period was comparable in both groups.

Figure 6 shows EEG spectral power in the delta range (1–4 Hz) across 24 h of spontaneous sleep–waking (A) and after 3 h of SD (B) in control and TNFα mice. During the 3 h SD period, TNFα mice showed significantly lower EEG spectral power in the theta range compared to controls (0.95 ± 0.17 mV^2^ vs. 2.19 ± 0.29 mV^2^, t = 3.875, *p* < 0.01). While TNFα mice exhibited a general decline in delta power during the 9 h baseline compared to controls (4.62 ± 0.76 mV^2^ vs. 5.76 ± 0.77 mV^2^, NS), this difference did not reach statistical significance. However, following SD, TNFα mice exhibited a significant reduction in high-delta activity during nonREM sleep in the initial 3 h of recovery compared to controls. This reduction persisted through 4–9 h of the recovery period (Figure 5B and Figure 6B).

4. Response to Pharmacologically induced sleep

**Figure 7:**
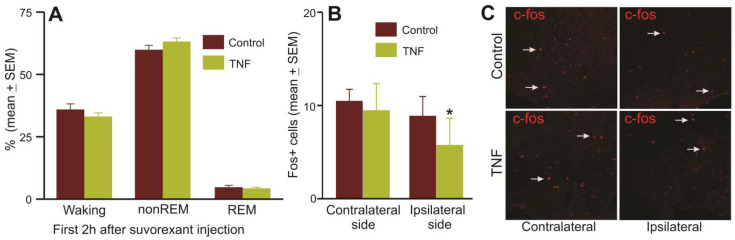
Fewer VLPO neurons express Fos-IR in response to suvorexant-induced sleep in TNFα mice. (**A**) The time (%, mean ± SEM) spent by control (*n* = 6) and TNFα (*n* = 9) mice as a group in waking, nonREM sleep, and REM sleep during the 2 h post-injection period after 30 mg/kg of suvorexant injection at the dark onset. Suvorexant produced comparable effects on sleep in both control and TNFα mice. (**B**) The number of VLPO cells expressing Fos-IR (mean ± SEM) in control (*n* = 6) and TNFα (*n* = 7) mice that were sacrificed after 2 h of suvorexant treatment. (**C**) Photomicrographs of sections through VLPO showing Fos-IR cells (arrowheads) on ipsilateral and contralateral sides of control and TNFα mice after suvorexant-induced sleep. While the numbers of VLPO cells expressing Fos-IR in control and TNFα mice were comparable on the contralateral side, fewer Fos-IR neurons were observed on the ipsilateral side of the TNFα mice, indicating that TNFα overproduction potentially caused dysfunction of local VLPO neurons. * *p* < 0.05 level of significance (Mann–Whitney test).

We compared sleep–wake amounts during 2 h post-suvorexant injection (30 mg/kg, IP) given at the beginning of the dark phase in control and TNFα mice to determine if mice with TNFα production in the POA-BF exhibited any deficit in responding to suvorexant-induced sleep and if it was due to dysfunction of the VLPO sleep-active neurons. The sleep–wake profiles of TNFα and control mice after suvorexant treatment and sleep-associated Fos-IR in VLPO are shown in Figure 7.

Both control and TNFα mice exhibited comparable levels of waking, nonREM sleep, and REM sleep during the 2 h period following suvorexant injection, indicating that control and TNFα mice responded similarly to suvorexant injection and that unilateral TNFα production in the POA-BF does not affect the ability of these mice to respond to suvorexant (Figure 7A). However, Fos-IR in these mice sacrificed after 2 h of suvorexant-induced sleep showed that while the numbers of VLPO cells expressing Fos-IR in these mice were comparable on the contralateral side, fewer Fos-IR cells were observed on the ipsilateral side of the TNFα mice compared to the control group (Figure 7B,C). This indicates that local TNFα production potentially caused dysfunction of VLPO neurons.

#### 3.2.3. Effects of Chronic TNFα Expression/Production in the POA-BF on Microglia Activation

**Figure 8:**
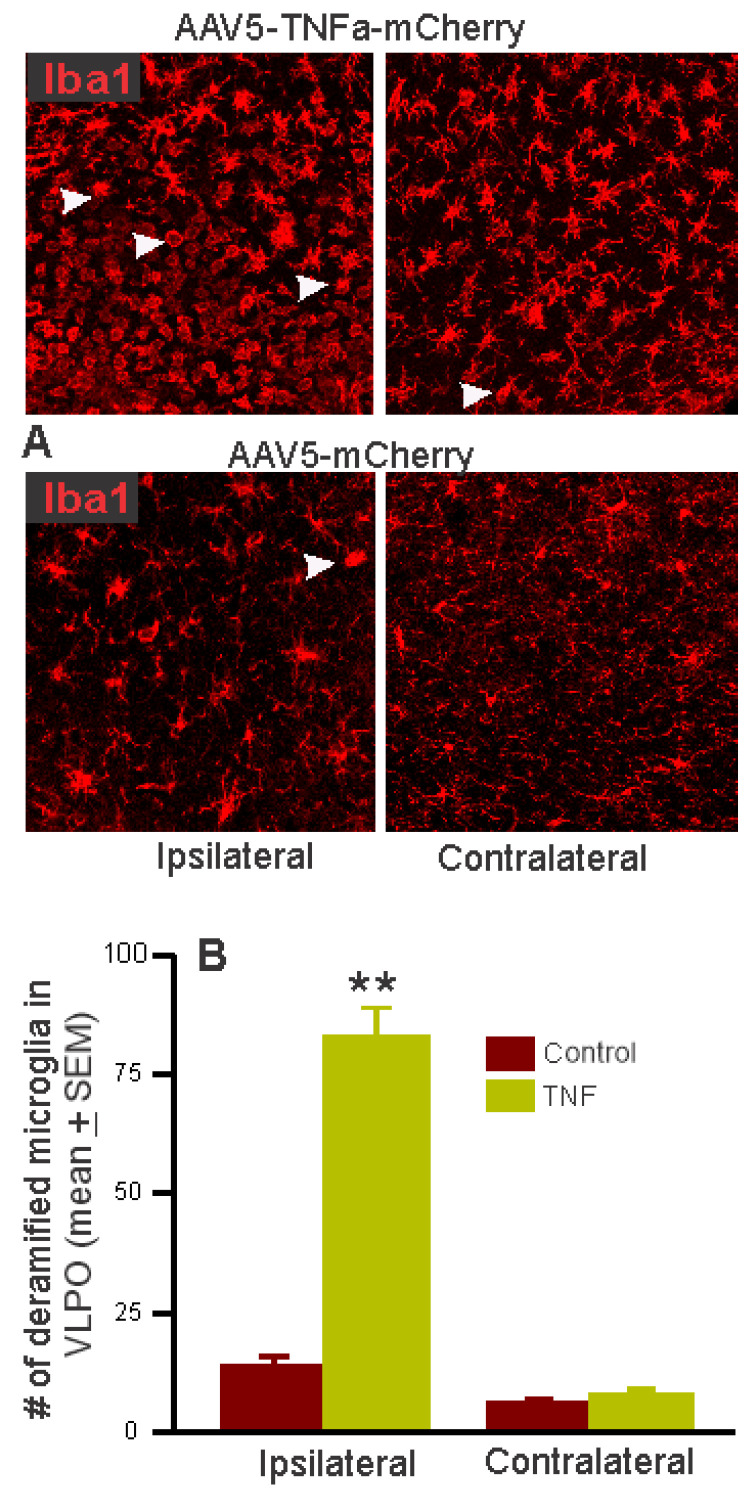
TNF-V activated microglia in the VLPO. (**A**) Photomicrographs of sections through VLPO showing Iba1+ and de-ramified cells (arrowheads) on ipsilateral and contralateral sides of control and TNFα mice. (**B**) The number of de-ramified Iba1 microglia. A dramatic increase in the number of activated or de-ramified microglia can be seen in the TNF-V injection site. ** *p* < 0.01 level of significance (Student *t*-test).

To evaluate the microglial response to inflammation caused by the expression of TNFα in astrocytes, we compared the number of de-ramified Iba1 stained cells in the VLPO region in control and TNFα mice. Compared to control, the number of de-ramified cells was significantly higher in TNFα mice (Figure 8). Only small and comparable numbers of de-ramified cells were observed on the contralateral side of TNFα mice and on the ipsilateral side of control mice.

We also compared the number of NeuN+ cells in the VLPO of control and TNFα mice to determine if TNFα overexpression caused any neuronal loss, which contributed to sleep–wake disruption. Both control and TNFα mice exhibited comparable numbers of NeuN+ cells both ipsilaterally (209 ± 38, *n* = 3 vs. 183 ± 12, *n* = 6, NS) and contralaterally (174 ± 35 vs. 201 ± 14; NS).

#### 3.2.4. Effects of Chronic TNFα Expression/Production in the POA-BF on Locomotor Activity and Motor Functions

(a) Rotarod performance: We used the rotarod test to assess if TNFα mice exhibited any deficit in sensorimotor coordination and motor learning. Both TNFα (*n* = 9) and control (*n* = 9) mice exhibited comparable latency to fall from the rotating rod or completed full passive rotation (Fall or complete passive rotation; 197 ± 18 vs. 185 ± 16 s, NS; Fall, 234 ± 38 vs. 227 ± 17 s, NS).

(b) Wire hang test: We evaluated the latency to fall in the wire hang test in control (*n* = 10) and TNFα (*n* = 9) mice to examine if mice with TNF in the POA-BF exhibit any deficit in motor functioning, particularly muscle strength, endurance, and grip strength. Compared to control mice (*n* = 10), TNF mice (*n* = 9) exhibited significantly reduced latency to fall off the wire lid or the ability to maintain their grip on the wire (113 ± 7 s vs. 81 ± 7 s, t = 3.22, *p* < 0.01).

(c) Open-field test: We used the open-field test to evaluate if TNFα (*n* = 9) and control (*n* = 9) mice exhibited any difference in general locomotor activity or exploratory behavior. Both control and TNFα mice exhibited comparable levels of locomotor and exploratory behavior during the 10 min of open-field test as evident from total distance traveled (28 ± 3 m vs. 27 ± 2), average speed (0.047 ± 0.004 m/s vs. 0.045 ± 0.003), total time mobile (556 ± 8 s vs. 550 ± 4 s), and number of entries to the central zone (32 ± 6 vs. 35 ± 3).

## 4. Discussion

While the detrimental effects of chronic central inflammation are widely known, its precise effects on sleep–wake regulatory systems and subsequent behavioral outcomes remain poorly understood. In this study, we used astrocyte-driven TNFα overproduction to induce a heightened local inflammatory milieu within the POA-BF and determine its impact on spontaneous and homeostatic sleep–wake regulatory functions, as well as on overall frailty as assessed by motor and physical performances. Using a carefully designed viral vector, we specifically targeted astrocytes to overexpress TNFα alongside a fluorescent reporter, mCherry, while excluding neurons and microglia. Our aim was to subject sleep- and wake-regulatory neural substrates within the POA-BF to increased levels of TNFα and downstream proinflammatory cytokines. Both TNFα and control vectors elicited robust mCherry expression exclusively in astrocytes-like cells within the POA-BF, confirming successful astrocytic transfection and TNFα production, thus validating the intended utility of this engineered viral vector.

We found that astrocytic TNFα overproduction led to local microglia activation, as indicated by a dramatic increase in de-ramified or activated microglia around the TNF-V injection site but not around the injection of the control virus or on the contralateral sides, indicative of a heightened inflammatory environment in the diffusion field of the TNF-V within the POA-BF. These mice also exhibited impaired sleep–wake functions, dysfunction of VLPO sleep-regulatory neurons, and impaired physical performance, notably:

(a) sleep disruption during the light phase as marked by (i) increased waking amounts with longer waking episodes; (ii) increased numbers of medium and decreased numbers of extended episodes of nonREM sleep, and a general decline in nonREM sleep amount (although marginal); and (iii) decreases in the amount and the number of REM episodes of >120 s;

(b) waking disturbance during the dark phase as marked by (i) a decline in the amount of waking with the increased number of shorter and decreased number of extended bouts of waking; (ii) frequent sleep intrusions by small and medium nonREM sleep episodes and an increased amount of nonREM sleep; and (iii) increases in the amount and number of REM sleep episodes;

(c) a decrease in theta power during waking;

(d) poor homeostatic response to sleep loss as indicated by decreased nonREM sleep rebound and lower nonREM sleep high-delta activity in response to 3 h of sleep deprivation;

(e) fewer Fos-IR neurons in the VLPO on the TNF-V injection side after suvorexant-induced sleep; and

(f) a decline in grip strength, leading to reduced ability to hang suspended on the wire.

To our knowledge, this is the first study where astrocytic TNFα overproduction has been used to trigger a chronic and heightened local inflammatory milieu within the POA-BF and determine its influences on sleep–waking and measures of frailty. The observed changes in sleep–waking and microglial activation in TNFα mice occurred specifically from astrocytic TNFα overproduction within the POA-BF and not from nonspecific effects triggered by vector injections, is evident from the following observations. Control vector (AAV5-GFAP-mCherry) into the POA-BF produced only insignificant sleep–wake changes over the four-week recording period, whereas injections of TNF-V (AAV5-GFAP-TNFα-mCherry) led to dose- and post-injection time-dependent changes in sleep–waking. After two weeks of injection, sleep–wake changes were largely comparable in all four groups (control and 3 doses of TNF-V). However, changes in sleep–wake architecture began to manifest after 3–4 weeks and became most pronounced by the end of the 5th week, presumably linked to the escalating levels of transgenic TNFα expression in astrocytes.

While TNF signaling plays a crucial role in regulating homeostatic functions, including sleep, neurogenesis, myelination, blood–brain barrier permeability, and synaptic plasticity [8,65,66], its excessive or inappropriate production can lead to inflammation, apoptosis, and a range of dysfunctions [67,68,69,70]. The findings of this study do support this notion, as mice with TNFα overproduction within the POA-BF exhibited disrupted spontaneous sleep–wake patterns and impaired homeostatic response to SD. Furthermore, while the number of NeuN+ cells was comparable in the TNFα and control groups, TNFα mice exhibited fewer Fos-IR neurons in the VLPO at the TNF-V injection site compared to the control group in response to suvorexant-induced sleep. These findings suggest that dysfunction of sleep- and wake-active neurons likely contributed to the observed sleep–wake impairments. These results underscore the role of heightened local inflammation in inducing neuronal dysfunction within the POA-BF, a critical component of the sleep–wake regulatory neuronal network.

It is worth noting that a relatively smaller number of Fos-IR neurons were observed in the VLPO after suvorexant-induced sleep. This could be because, firstly, nearly 50% of VLPO neurons are wake-active and would be inhibited by suvorexant [71,72]; and secondly, inflammatory molecules like IL-1 predominantly exert inhibitory effects on POA neurons [73,74]. The mechanisms by which chronic inflammation affects the physiology of wake- and sleep-active neuronal groups remain poorly understood and require further studies.

TNFα mice exhibited pronounced microglial activation around the injection site, indicating that the observed sleep–wake disruptions were likely triggered by TNFα production in transfected astrocytes, affecting local neuronal and glial populations within the POA-BF. This process likely led to the activation of microglia and subsequent release of more inflammatory molecules, intensifying the levels of inflammation and consequent sleep–wake and behavioral impairments. Some glial activation was also observed around the injection site of control mice, which could be due to needle-induced injury or the expression of high-titer AAV5/mCherry construct [75]. It is pertinent to note that in this study, we employed the GFAP promotor to broadly target astrocytes, assuming that TNFα production would prompt a proinflammatory state. However, astrocytes are heterogeneous [76,77,78], and it remains uncertain which subtypes undergo this transition. Further studies are needed to gain insights into how TNFα affects astrocytes themselves and the specific roles of astrocytes in regulating sleep–waking, particularly in regions that accommodate both sleep- and wake-active neurons like the VLPO-BF.

Evidence suggests that aging is associated with chronic low-grade inflammation and disruptions in sleep–wake patterns (see Introduction, Section 1). Our study demonstrates that heightened chronic inflammation in the POA-BF of young mice induces aging-like changes in sleep–wake organization. Notably, disrupted sleep during the light phase, when animals are primarily asleep, and waking disturbances during the dark phase, when animals are mainly awake, suggest that chronic TNFα overproduction detrimentally affects both sleep and wake regulatory systems within the POA-BF. However, despite an impaired sleep-regulatory system, suvorexant-induced sleep in these mice was comparable to that of the control group. It is not surprising and indicates that the suvorexant’s inhibition of wake-promoting systems throughout the neuroaxis overrides the dysfunction of a smaller group of sleep-promoting neurons affected by TNFα. However, it is important to note that animals were sacrificed for immunohistochemistry 2 h post-suvorexant injection, and a mere 2 h sleep recording may not conclusively ascertain that TNFα mice did not exhibit an attenuated response to suvorexant. Overall, our findings support a hypothesis that chronic neuroinflammation contributes to sleep–wake dysfunction in aging. Our findings further suggest that dysfunction of sleep- and wake-regulatory mechanisms within the POA-BF due to neuroinflammation may be a critical component of sleep–wake disturbances in aging.

TNFα mice did not exhibit signs of sickness behavior, or reduced locomotor/exploratory activity, and stereotypical/anxiety-like behaviors in the open-field test, which are typically associated with systemically induced inflammation [52,53,54,79]. TNFα mice performed well on the rotarod test, indicating no deficits in motor coordination or muscle strength and endurance [55,56]. However, they performed significantly poorly on the wire hang test, suggesting compromised grip strength and neuromuscular function likely influenced by chronic inflammation in the POA-BF and/or associated sleep disruption [57,58,80].

In humans, reduced grip strength is linked to functional decline and elevated markers of systemic inflammation [81,82], which correlates with reduced skeletal muscle strength and mass [83,84]. Although we did not measure systemic proinflammatory markers in TNFα mice, evidence suggests that central transgenic overproduction of TNFα in mice may not elevate TNFα levels in serum [85], implying that central and peripheral inflammation in TNFα mice may not reach levels detrimental to open-field and rotarod performance. However, TNFα mice exhibited significant sleep impairment, likely comparable to chronic sleep disruption associated with systemic and central accumulation of proinflammatory molecules, including TNFα [86,87], contributing to reduced grip strength. Our findings are consistent with studies on inflammation models, indicating the wire hang test’s greater sensitivity compared to the rotarod test in assessing physical performance [88]. It is plausible that TNFα mice may develop further deficits as sleep disruption worsens over time.

Limitations: While not limiting to the findings or conclusions derived, it is important to acknowledge certain limitations of this study.

(1) No direct measurement of TNFα in the POA-BF. We did not measure TNFα protein levels directly. Instead, we relied on indirect markers of TNFα production and inflammation, such as tracking mCherry-positive cells as an indicator of transgenic TNFα expression, and quantifying de-ramified microglia as a marker of heightened inflammation and increased TNFα production and activity.

(2) Limited validation of TNFα-producing cells. In this study, we examined the colocalization of mCherry, the transfection marker, mainly with NeuN+ (neurons) and Iba1+ (microglia) cells, and in a limited number of animals with GFAP+ cells (astrocytes). We found minimal to no mCherry expression in neurons and microglia, but predominant expression in GFAP+ cells. However, some mCherry-expressing cells were not GFAP+, which is not completely unexpected. While several proteins are selectively expressed by astrocytes, none are expressed in all astrocytic subtypes, and studies show that GFAP immunolabeling only accounts for about 15% of the total astrocyte volume and up to 40% of astrocytes may not express GFAP at levels detectable by standard staining [89,90,91]. Given these findings and that we usedGFAP promoter to drive TNFα-mCherry expression, it is reasonable to infer that astrocytes were predominantly transfected, making them the primary source of TNFα in mCherry-positive cells.

Methodological considerations: Existing mice models of TNFα-induced inflammation have provided valuable insights into its effects on behavior and physiology. Peripheral TNF elevation via repeated IP injections or subcutaneous (SC) osmotic pump delivery has been widely utilized. While effective in inducing acute effects, maintaining chronic TNFα elevation in the brain remains challenging [85].

Alternatively, transgenic approaches for TNFα expression offer persistent and prolonged neuroinflammation. Injections of AAV-TNF into the cerebral ventricle or specific brain regions induce mild TNF elevation or microglia activation in targeted areas without affecting plasma TNF levels, suggesting region-specific effects on behavior [85]. However, the use of CMV or CAG promoters in viral transfections lacks cell specificity in TNFα expression.

To overcome these limitations, we developed a novel viral construct selectively targeting astrocytes to induce local chronic TNFα-mediated neuroinflammation in mice. Using this approach, we investigated the effects of chronic neuroinflammation in the POA-BF on sleep and behavioral performance. This study validates the utility of our model for examining the impact of chronic local inflammation in various brain regions across vertebrate species.

## 5. Conclusions

This study found that astrocytic TNFα overproduction led to increased microglial activation, indicating a heightened inflammatory milieu within the POA-BF. These mice also exhibited aging-like changes in sleep–wake organization and physical performance, including (a) disruptions in both sleep and waking during light and dark phases and a reduced ability to compensate for sleep loss; (b) dysfunction of VLPO sleep-active neurons; and (c) a decline in grip strength indicating a compromised neuromuscular function. These findings suggest that inflammation-induced dysfunction of sleep- and wake-regulatory mechanisms within the POA-BF may be a critical component of sleep–wake disturbances in aging. However, further studies are needed to understand the mechanisms through which chronic inflammation affects the physiology of wake- and sleep-active neuronal groups in the POA-BF.

## Figures and Tables

**Table 1:** Sleep–wake profiles of control and TNFα groups: within-group comparison.

**Light Phase**	**Week 2**	**Week 3**	**Week 4**	**Week 5**	**F Value**	***p* Value**
**Control vector**						
Waking	34.2 ± 1.1	34.1 ± 1.0	33.2 ± 1.3	36.3 ± 2.1	F_9,3_ = 1.07	0.38
nonREM sleep	58.6 ± 0.9	58.8 ± 1.0	59.7 ± 1.3	59.1 ± 1.5	0.67	0.58
REM sleep	7.2 ± 0.4	7.0 ± 0.2	7.2 ± 0.2	6.9 ± 0.2	0	0.76
**TNF vector-Low**						
Waking	36.7 ± 2.4	36.6 ± 3.6	33.6 ± 1.7	34.0 ± 1.5	F_5,3_ = 0.69	0.57
nonREM sleep	61.0 ± 1.3	60.2 ± 1.9	57.65 ± 3.2	56.5 ± 2.4	1.26	0.32
REM sleep	6.9 ± 0.4	5.8 ± 0.7	6.2 ± 0.4	5.0 ± 0.7 *^,$^	5.85	<0.01
**TNF-vector Medium**						
Waking	33.5 ± 2.3	35.7 ± 2.4	34.2 ± 1.9	33.7 ± 1.9	F_5,3_ = 0.66	0.58
nonREM sleep	58.8 ± 2.0	57.4 ± 2.1	59.4 ± 1.6	60.7 ± 1.6	2.68	0.08
REM sleep	7.7 ± 0.4	6.9 ± 0.5	6.4 ± 0.6	5.7 ± 0.7 *	3.76	<0.05
**TNF-vector High**						
Waking	36.5 ± 1.2	38.8 ± 2.1	41.1 ± 2.4 *	40.9 ± 2.6 *	F_8,3_ = 4.86	0.009
nonREM sleep	56.4 ± 1.3	55.4 ± 2.1	54.5 ± 2.3	55.3 ± 2.4	0.77	0.51
REM sleep	7.1 ± 0.4	5.8 ± 0.3 **	4.3 ± 0.3 **^,$$^	3.9 ± 0.3 **^,$$^	34.95	<0.001
**Dark Phase**	**Week 2**	**Week 3**	**Week 4**	**Week 5**	**F Value**	***p* Value**
**Control vector**						
Waking	65.4 ± 3.6	72.0 ± 3.0	71.9 ± 2.3	71.2 ± 2.3	3.12	0.04
nonREM sleep	32.4 ± 3.3	26.1 ± 2.8	26.6 ± 2.2	27.0 ± 2.3	3.18	0.04
REM sleep	2.1 ± 0.4	1.9 ± 0.3	1.5 ± 0.2	1.7 ± 0.2	1.62	0.2
**TNF vector-Low**						
Waking	60.2 ± 1.9	65.0 ± 3.4	67.1 ± 3.0	66.4 ± 3.2	2.41	0.11
nonREM sleep	36.8 ± 1.7	32.5 ± 3.1	30.6 ± 2.8	31.3 ± 2.8	2.35	0.11
REM sleep	3.0 ± 0.4	2.5 ± 0.3	2.3 ± 0.4	2.3 ± 0.5	1.85	0.18
**TNF-vector Medium**						
Waking	66.0 ± 2.3	65.9 ± 4.2	63.9 ± 5.1	62.3 ± 4.4	2.37	0.11
nonREM sleep	31.6 ± 3.2	31.9 ± 3.9	33.9 ± 4.8	35.5 ± 4.1	2.67	0.09
REM sleep	2.4 ± 0.3	2.2 ± 0.3	2.3 ± 0.3	2.2 ± 0.3	0.22	0.87
**TNF-vector High**						
Waking	64.3 ± 2.0	58.9 ± 2.5 *	52.9 ± 2.7 *^,$^	51.8 ± 2.14 *^,$^	28.97	<0.001
nonREM sleep	32.6 ± 1.8	37.3 ± 2.2 **	43.5 ± 2.5 **^,$$^	45.2 ± 2.0 **^,$$^	39.3	<0.001
REM sleep	3.1 ± 0.3	3.7 ± 0.5 **	3.6 ± 0.4	2.98 ± 0.4	3.76	<0.05

One-Way repeated measure ANOVA; * and read as *, compared to week-2; $, compared to week-3; *, $, *p* < 0.05, **, $$, *p* < 0.01 level of significance.

## Data Availability

The result section sufficiently describes the data. Further details or the raw data or analysis are available for sharing/presentation upon request to the corresponding author.

## Supplementary Materials

The following supporting information can be downloaded at: https://www.mdpi.com/article/10.3390/cells13110894/s1, Figure S1: TNFα-cherry expression was astrocyte-specific. Photomicrographs of representative histological sections showing mCherry expression and its colocalization with GFAP+ cells (anti GFAP antibody ab7260, 1:1000; Abcam) after AAV5-GFAP-TNFα-mCherry injections in 3 animals. This figure shows that mCherry expression predominantly coincided with GFAP+ cells, indicating astrocyte-specificity. However, some mCherry-expressing cells were not GFAP+, which is not completely unexpected. While several proteins are selectively expressed by astrocytes, none are expressed in all astrocytic subtypes, and studies show that GFAP immunolabeling only accounts for about 15% of the total astrocyte volume and up to 40% of astrocytes may not express GFAP at levels detectable by standard staining [89,90,91]. Importantly, mCherry expression did not colocalize with NeuN+ (neurons) or Iba1+ (microglia) cells, but rather with astrocyte-like cells (see Figure 2). This aligns with the astrocyte specificity of the GFAP promoter used in our viral vector (48,49]. Overall, our findings and previous studies support that TNFα-mCherry expression was likely confined to astrocytes, supporting astrocyte-specific targeting of this construct.
