# Supplementary material for: Chronic Astrocytic TNFα Production in the Preoptic-Basal Forebrain Causes Aging-like Sleep–Wake Disturbances in Young Mice
Source: Cells. 2024 May 22;13(11):894. doi: 10.3390/cells13110894 (PMC11171867; doi:10.3390/cells13110894)
Supplement: Supplementary file 1 [file cells-13-00894-s001.zip › cells-2970072-supplementary.pdf]

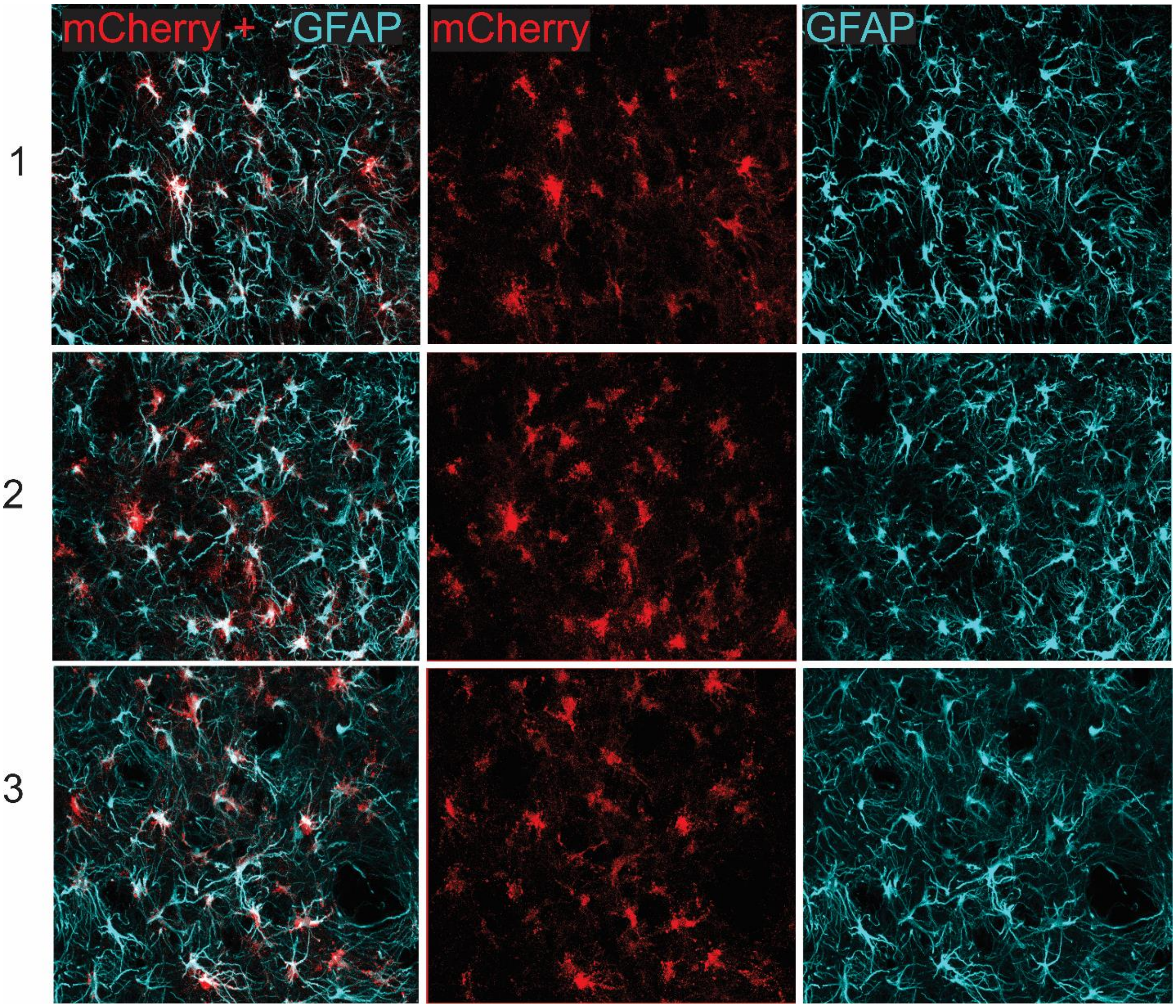

Figure S1: TNFα-cherry expression was astrocyte-specific. Photomicrographs of representative histological sections showing mCherry expression and its colocalization with GFAP+ cells (anti GFAP antibody ab7260, 1:1000; Abcam) after AAV5-GFAP-TNFα-mCherry injections in 3 animals. This figure shows that mCherry expression predominantly coincided with GFAP+ cells, indicating astrocyte-specificity. However, some mCherry-expressing cells were not GFAP+, which is not completely unexpected. While several proteins are selectively expressed by astrocytes, none are expressed in all astrocytic subtypes, and studies show that GFAP immunolabeling only accounts for about 15% of the total astrocyte volume and up to 40% of astrocytes may not express GFAP at levels detectable by standard staining [89-91]. Importantly, mCherry expression did not colocalize with NeuN+ (neurons) or Iba1+ (microglia) cells, but rather with astrocyte-like cells (see Figure 2). This aligns with the astrocyte specificity of the GFAP promoter used in our viral vector (48,49]. Overall, our findings and previous studies support that TNFα-mCherry expression was likely confined to astrocytes, supporting astrocyte-specific targeting of this construct.
